# Control of articular synovitis for bone and cartilage regeneration in rheumatoid arthritis

**DOI:** 10.1186/s41232-018-0064-y

**Published:** 2018-04-16

**Authors:** Hiromu Ito, Furu Moritoshi, Motomu Hashimoto, Masao Tanaka, Shuichi Matsuda

**Affiliations:** 1 The Department of Orthopaedic Surgery, Kyoto University Graduate School of Medicine, 54 Kawahara-cho, Shogoin, Sakyo, Kyoto, 606-8507 Japan; 2 The Department of Advanced Medicine for Rheumatic Diseases, Kyoto University Graduate School of Medicine, Kyoto, Japan

**Keywords:** Rheumatoid arthritis, Joint destruction, Synovitis, Articular cartilage, Regeneration

## Abstract

**Background:**

Rheumatoid arthritis is an autoimmune inflammatory disease, the specific feature of which is progressive joint destruction induced by synovitis. The universal consensus is that alleviation of the synovitis is essential to prevent joint destruction and achieve clinical remission.

**Main text:**

We have shown that not only achieving but also maintaining remission is crucial to prevent the progression of joint destruction. Although regeneration of the damaged joints is considered very rare, accumulating evidence shows that it actually occurs in routine clinical practice as a result of strong inhibition of synovitis using highly potent medications. Oral and intravenous medications affect the whole body, but to promote joint regeneration in a particular joint, two potent options are intra-articular steroid injection and synovectomy.

**Conclusion:**

In situations where strong inhibition of synovitis combined with self-regeneration cannot repair severe joint destruction, regenerative medicine may in the future play a crucial role in the regeneration of damaged joints.

## Background

### RA pathology: joint destruction and synovitis

Rheumatoid arthritis (RA) is characterized by spontaneous progressive joint destruction that is predominantly caused by persistent, chronic synovitis in the joint [1]. Treatment with disease-modifying anti-rheumatic drugs (DMARDs) improves RA disease activities, but even with the best currently available treatment, residual disease activity can induce inflammatory joint damage such as erosion and joint-space narrowing that can be progressive and irreversible and that results in functional impairment [2–4].

The loss of the articular cartilage in RA is evident on X-ray as joint-space narrowing, but in most cases, erosion and joint-space narrowing progress coordinately. Therefore, most studies show combined data for erosion and joint-space narrowing as exemplified by one of the most widely used joint destruction scores, the modified total Sharp score [5–7]. Moreover, although they do not progress separately, it is considered that erosion and joint-space narrowing can affect one another. Because joint destruction is mostly irreversible and directly causes joint pain and functional disability, a key target of treatment is prevention of joint destruction, and it is a fundamental rule of treatment that results should be determined, at least partially, by how well the treatment can prevent joint destruction [8].

As indicated above, synovitis is a fundamental clinical and pathological feature of RA and is largely responsible for the disease-associated joint destruction. Therefore, the basic strategy of treatment is to inhibit or alleviate synovitis; numerous clinical and basic studies have shown that this can prevent joint destruction. Most studies, guidelines, and recommendations have suggested that prevention of joint destruction can be achieved by decreasing disease activity and maintaining this lower activity as remission [9, 10]. Moreover, to suppress the progression of joint destruction, alleviation of synovitis should be achieved as early as possible within the (therapeutic) *window of opportunity* [11, 12].

### Clinical remission and joint destruction

The main goal of RA treatment is to suppress disease activity as early in the disease process as possible, thereby achieving clinical remission and preventing radiographic damage and disability. Several sets of criteria to define clinical remission in RA have been proposed and applied, starting with the 1981 American College of Rheumatology (ACR) definition of remission [13], followed by the definition of remission as a disease activity score of less than 1.6 based on 44 joints (DAS44) [14], later modified to a score of less than 2.6 involving 28 joints (DAS28) [15], a clinical disease activity index (CDAI) of less than 2.8 [16], and a simplified disease activity index (SDAI) of less than 3.3 [17]. More recently, the ACR and the European League Against Rheumatism (EULAR) collaborated to propose that remission in RA can be defined either according to the remission criteria of both the CDAI and the SDAI or the new Boolean-based set of criteria (ACR/EULAR remission criterion) [18].

Treat to target (T2T) is considered a key strategy in the induction of remission in individual RA patients [19]. DAS28 remission is a feasible goal in daily clinical practice with the application of a T2T strategy of early and intensive treatment of patients with early RA, which leads to high remission rates [20] and limited radiographic progression after 1 year of follow-up [21]. However, clinical trials have demonstrated that some patients with RA in remission defined by DAS28 showed residual joint swelling and radiographic progression compared with patients in remission defined by ACR/EULAR. However, the ACR/EULAR remission criteria are difficult to achieve in patients with established RA. It is unclear which criteria should be used and how often clinical remission can be achieved in daily clinical practice.

### Sustained clinical remission contributes to functional remission and less radiological progression

On the basis of these considerations, we conducted a retrospective longitudinal study to investigate whether sustained clinical remission would reduce functional disability and radiological progression, to identify which remission criteria best reflected functional and radiological remission, and how often clinical remission should be achieved in daily clinical practice. The results of this study were partially described in an article in the official journal of the Japanese Orthopaedic Association [22].

#### Materials and methods

In 2012, we enrolled 384 patients from the Kyoto University Rheumatoid Arthritis Management Alliance (KURAMA) cohort [23], and complete datasets for 170 of these patients, with both more than 6 months of follow-up and with more than three visits during follow-up, were used in this study. The data collected included age, sex, disease duration, Steinbrocker class, Steinbrocker stage, swollen joint count based on assessment of 28 joints (SJC28), tender joint count based on assessment of 28 joints (TJC28), the presence of rheumatoid factor (RF) and/or anti-citrullinated protein antibodies, C-reactive protein level, erythrocyte sedimentation rate (ESR), score on the Health Assessment Questionnaire disability index (HAQ-DI) [24], the patient’s assessment of pain measured using a 100-mm visual analogue scale (VAS), and global assessments of disease activity by evaluators (EGA) and patients (PGA). The radiographs were scored according to the van der Heijde-modified Sharp scoring method by two trained physicians blinded to the sequence of the radiographs [6]. The change in the Sharp/van der Heijde score (SHS) during follow-up was the main outcome of the study and was divided by the years of follow-up to calculate the annual rate of change. Patients with more than 1 unit change in SHS per year were classified as “progressors” [25]. Patients with 5 or more unit change in SHS per year were classified as showing rapid radiographic progression (RRP). Four different remission criteria were evaluated in this study: DAS28–ESR calculated including ESR (mm/h), TJC28, SJC28, and the PGA. Remission was defined as reported previously [16, 17]. The rate of remission maintenance was calculated by dividing the length of time for each remission by the number of patient visits throughout the follow-up period. “Complete sustained remission” was defined as a maintenance rate of 100%, “nearly sustained remission” was defined as a maintenance rate of 50% or more, “incomplete sustained remission” was defined as a maintenance rate of less than 50%, and “no remission” was defined as a maintenance rate of 0%.

#### Results

**Fig. 1:**
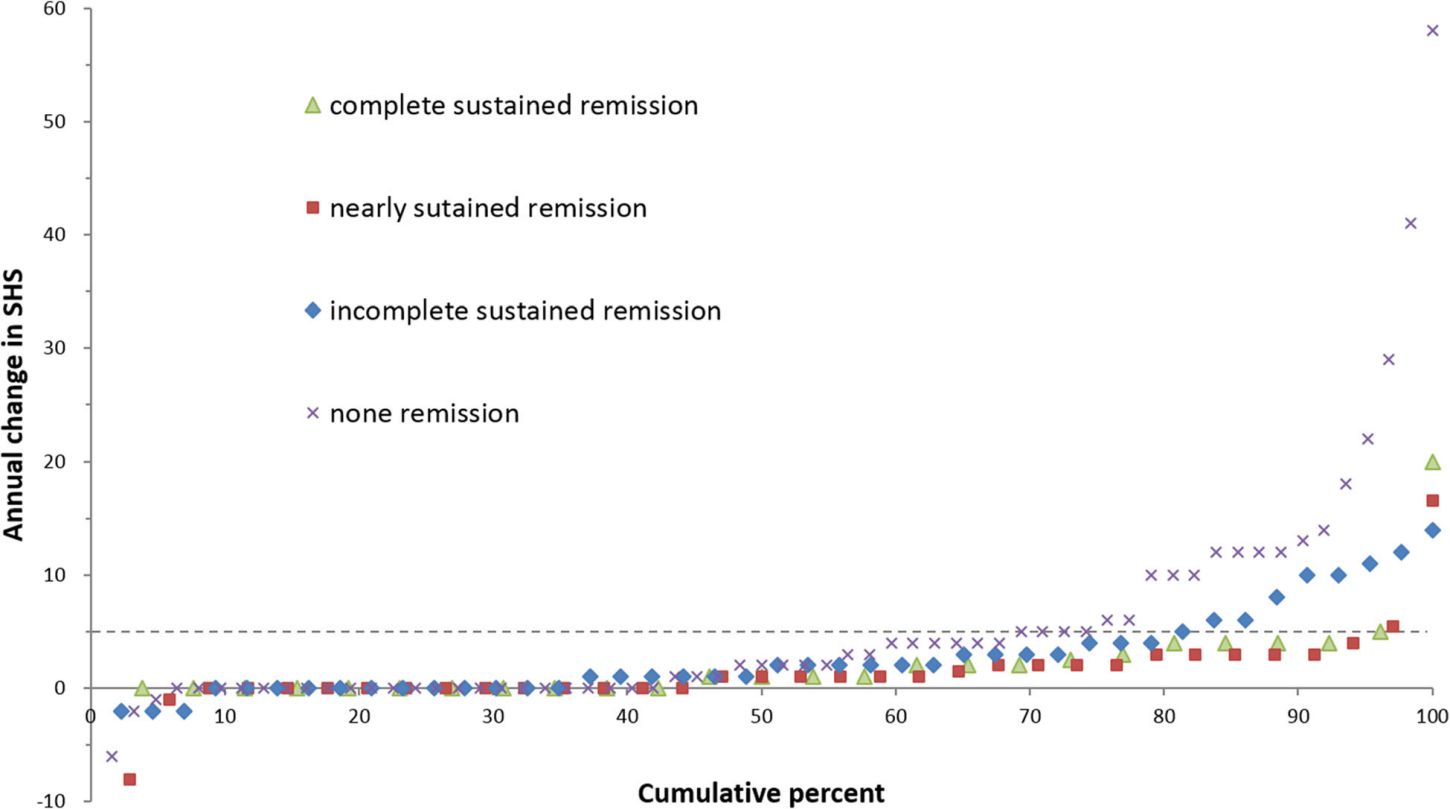
Cumulative probability plot for patients in sustained remission according to DAS28-ESR criterion during follow-up period

**Fig. 2:**
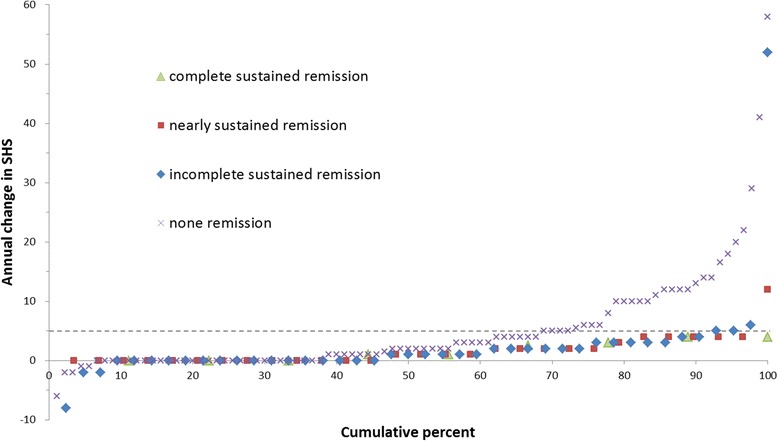
Cumulative probability plot for patients in sustained remission according to CDAI criterion during follow-up period

**Fig. 3:**
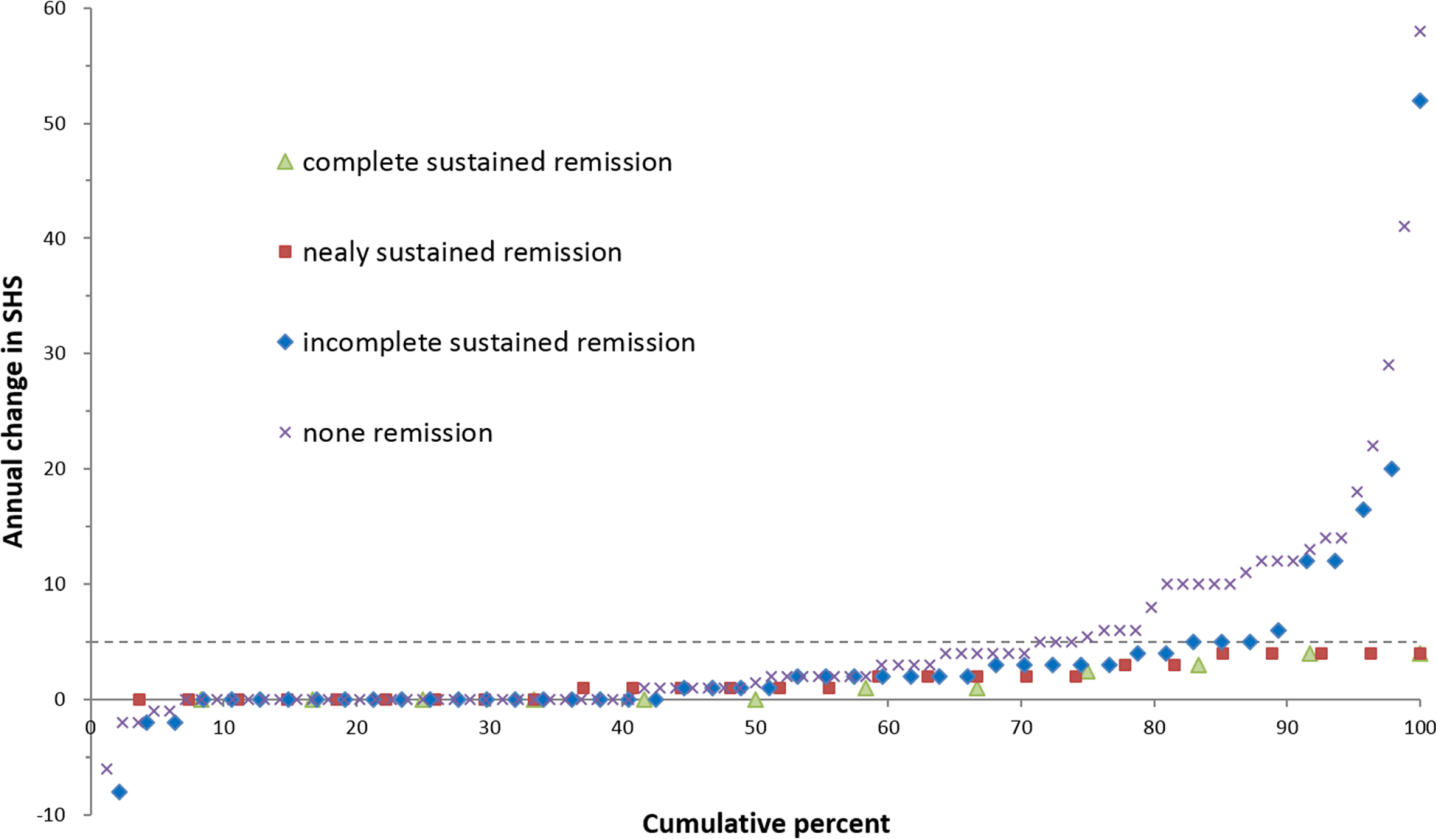
Cumulative probability plot for patients in sustained remission according to SDAI criterion during follow-up period

**Fig. 4:**
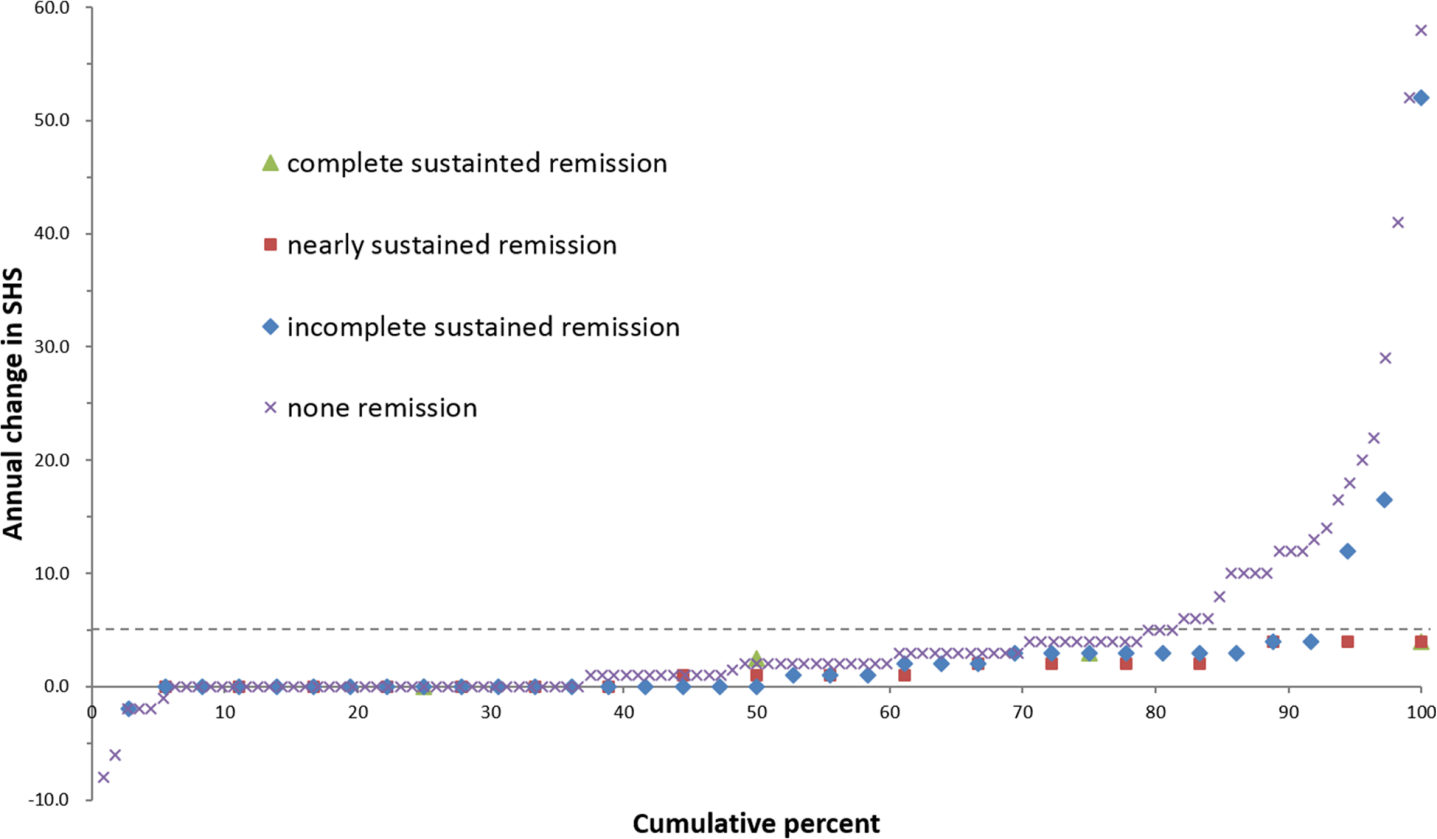
Cumulative probability plot for patients in sustained remission according to Boolean based remission criterion during follow-up period

**Fig. 5:**
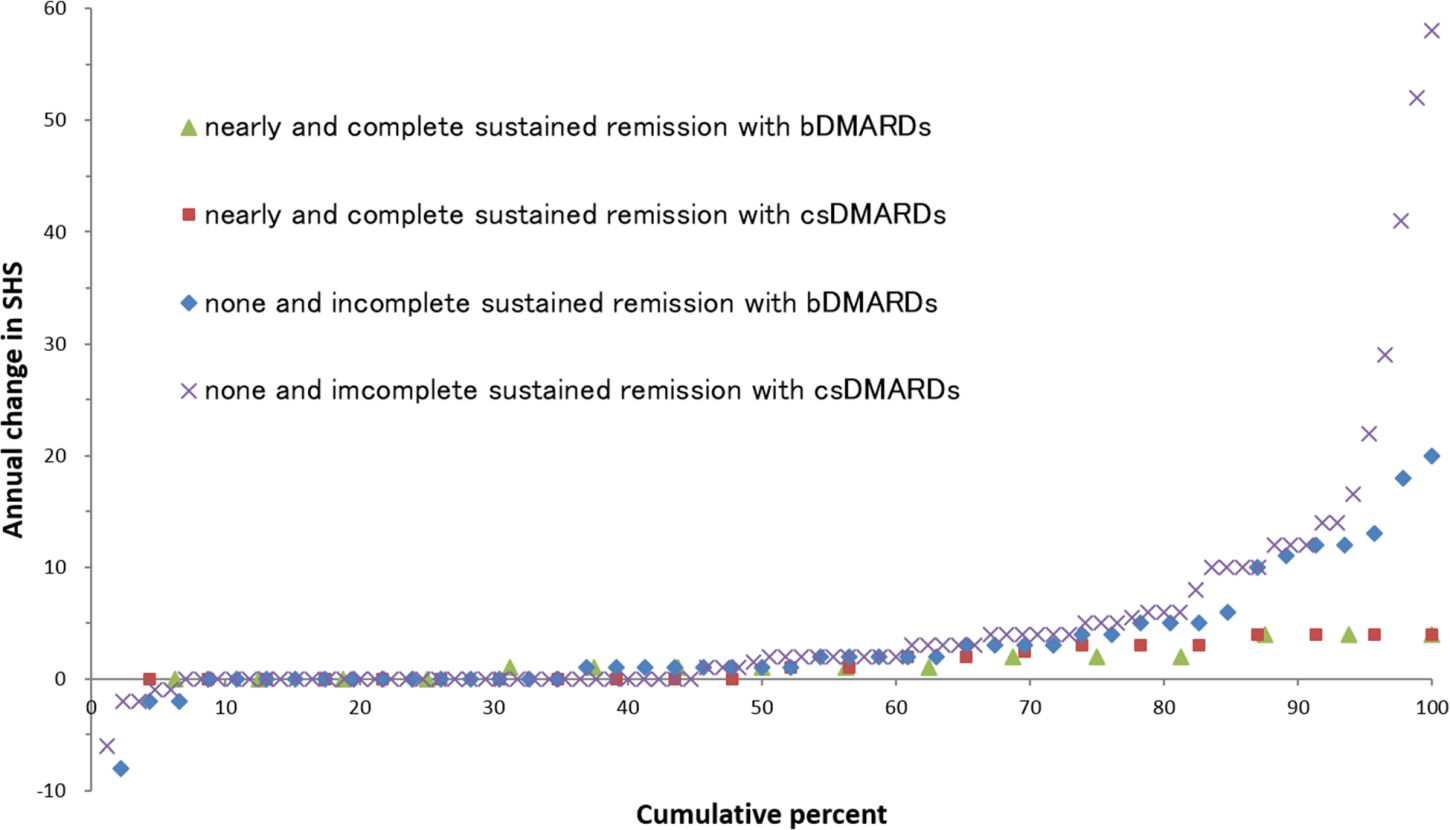
Cumulative probability plot for patients in sustained remission according to SDAI criterion during follow-up period compared bDMARDs with csDMARDs

The demographic characteristics of the patients are shown in Table 1. Among the 170 patients, the mean (SD) maintenance rates of clinical remission were 38.4% (38.3%) using DAS28, 23.0% (31.5%) using CDAI, 25.0% (32.7%) using SDAI, and 15.0% (25.7%) using Boolean-based remission criteria (Table 2). To determine whether biological DMARDs (bDMARDs) maintained clinical remission better than conventional synthetic DMARDs (csDMARDs), we compared the maintenance rates for each remission definition between the 62 patients treated with bDMARDs and the 108 patients treated with csDMARDs. The maintenance rate of remission defined according to DAS28–ESR was higher with bDMARDs than with csDMARDs (bDMARDs: mean 48.9%, csDMARDs: mean 32.4%; *P* < 0.01). However, there were no significant differences between bDMARDs and csDMARDs in the maintenance rates of remission defined according to CDAI, SDAI, and Boolean-based criteria. Analysis of functional impairment represented by HAQ-DI indicated that sustained clinical remission contributed to functional remission of RA (Table 3). The radiographic progression rates of patients as assessed by DAS28–ESR, SDAI, CDAI, and Boolean-based remission criteria are illustrated in Table 4. The annual change in SHS and a cumulative probability plot showing the individual data for all patients are presented in Figs. 1, 2, 3, and 4. There were fewer radiological progressors in the complete sustained remission and nearly sustained remission groups than in the incomplete sustained remission and no remission groups, for all definitions of remission. The number of progressors was approximately equivalent in the complete and nearly sustained remission groups as assessed by all criteria, although no patients in either the complete or nearly sustained remission groups assessed by either SDAI or Boolean-based criteria were classified as RRP. To determine whether biological bDMARDs reduced radiological progression better than csDMARDs, we compared the annual change in SHS in the 62 patients treated with bDMARDs and the 108 patients treated with csDMARDs (Fig. 5). No instance of RRP was observed for patients treated with either bDMARDs or csDMARDs in the complete and nearly sustained remission groups as defined by the SDAI criteria. However, RRP was observed with both treatments in the groups with incomplete sustained or no remission defined by the SDAI criteria.

**Table 1:** Characteristics of the patient population

	Mean ± SD or *n* (percent)	Median (range)
Age, years	62.7 ± 12.4	64.5 (31~85)
Disease duration, years	13.6 ± 11.5	11.25 (0.4~64.3)
Women	140 patients (82.4%)	
Steinbrocker‘s stage, I/II/III/IV	23 (13.5%)/53 (31.2%)/27 (15.9%)/67 (39.4%)	
Steinbrocker’s class, 1/2/3/4	41 (24.1%)/103 (60.6%)/25 (14.7%)/1 (0.01%)	
Rheumatoid factor positive	133 (78.2%)	
Anti-citrullinated protein antibody positive	135 (79.4%)	
C-reactive protein, mg/dl	0.61 ± 1.13	0.1 (0~5.4)
Erythrocyte sedimentation rate, mm/h	25.4 ± 21.1	18.5 (0~117)
Tender joint count, 0 to 28joints	1.1 ± 2.0	0 (0~14)
Swollen joint count, 0 to 28joints	1.2 ± 1.7	0 (0~9)
Patient’s global assessment, 0 to 100 mm	36.8 ± 26.3	32 (1~100)
Evaluator’s global assessment, 0 to 100 mm	14.6 ± 15.1	10 (0~73)
Disease activity score in 28 joints at endpoint	3.05 ± 1.22	2.94 (0.00~5.90)
Simplified disease activity index at endpoint	7.99 ± 6.51	5.90 (0.20~30.40)
Clinical disease activity index at endpoint	7.37 ± 5.97	5.75 (0.20~28.00)
Health Assessment Questionnaire disability index, 0 to 3	0.90 ± 0.76	0.8125 (0~3)
Sharp/van der Heijde score at baseline	109.5 ± 101.1	77.5 (1~398)
Sharp/van der Heijde score at endpoint	112.7 ± 101.6	79.5 (2~401)
Annual change of Sharp/van der Heijde score	3.6 ± 7.9	1 (− 8~58)
Use of glucocorticoid	76 (44.7%)	
Use of methotrexate	141 (70.6%)	
Use of biologics	62 (36.5%)	
TNF inhibitors	40 (23.5%)	
Tocilizumab	12 (7.1%)	
Abatacept	10 (5.9%)	

Data representing the demographic characteristics of the 170 patients. Data are presented as means ± S.D. or *n* (percent) and median (range)

**Table 2:** Proportions of patients who sustained clinical remission according to each criterion during follow-up period

	DAS28-ESR	CDAI	SDAI	Boolean-based
Mean (SD) maintain rate of remission, %	38.4 (38.3)	23.0 (31.5)	25.0 (32.7)	15.0 (25.7)
No. (%) of complete sustained remission	26 (15.3)	9 (5.3)	12 (7.1)	4 (2.4)
No. (%) of nearly sustained remission	39 (22.9)	29 (17.1)	27 (15.9)	18 (10.6)
No. (%) of incomplete sustained remission	43 (25.3)	42 (24.7)	47 (27.6)	36 (21.2)
No. (%) of none remission	62 (36.5)	90 (52.9)	84 (49.4)	112 (65.9)

Data represent the proportions of RA patients who sustained clinical remission according to each criterion during follow-up period. Complete sustained remission was defined as maintain rate of 100%. Nearly sustained remission was defined as maintain rate of 50% or more. Incomplete sustained remission was defined as maintain rate of less than 50%. None remission was defined as maintain rate of 0%. Boolean-based is one of the ACR/EULAR remission criteria

*DAS28–ESR* disease activity score based on 28 joint count and erythrocyte segmentation rate, *CDAI* clinical disease activity index, *SDAI* simplified disease activity index

**Table 3:** Functional disability of patients who sustained clinical remission according to each criterion during follow-up period

		DAS28-ESR	CDAI	SDAI	Boolean-based
Complete sustained remission	Mean (SD) HAQ-DI No. (%) of functional remission	0.37 (0.14) 20 (76.9)	0.36 (0.25) 7 (77.8)	0.43 (0.22) 9 (75.0)	0.22 (0.38) 4 (100)
Nearly sustained remission	Mean (SD) HAQ-DI No. (%) of functional remission	0.65 (0.12) 22 (56.4)	0.30 (0.13) 25 (86.2)	0.33 (0.14) 22 (81.5)	0.39 (0.18) 14 (77.8)
Incomplete sustained remission	Mean (SD) HAQ-DI No. (%) of functional remission	0.93 (0.12) 15 (34.9)	0.70 (0.12) 18 (42.9)	0.71 (0.11) 21 (44.7)	0.43 (0.12) 23 (63.9)
None remission	Mean (SD) HAQ-DI No. (%) of functional remission	1.26 (0.09) 14 (22.6)	1.24 (0.07) 21 (23.3)	1.26 (0.07) 19 (22.6)	1.15 (0.06) 30 (26.8)

Functional disability was assessed by HAQ-DI at endpoint. Complete sustained remission was defined as maintain rate of 100%. Nearly sustained remission was defined as maintain rate of 50% and more. Incomplete sustained remission was defined as maintain rate of less than 50%. None remission was defined as maintain rate of 0%. Functional remission was defined as HAQ-DI < 0.5

*HAQ-DI* health assessment questionnaire disability index

**Table 4:** Radiographic progression of patients who sustained clinical remission according to each criterion during follow-up period

			DAS28-ESR	CDAI	SDAI	Boolean-based
Complete sustained remission	Annual change in SHS	Mean (SD)	2.2 (4.0) 0–20	1.7 (1.7) 0–4	1.3 (1.6) 0–4	2.4 (1.7) 0–4
		range				
	No. (percent) of RRP		2 (7.7)	0 (0)	0 (0)	0 (0)
Nearly sustained remission	Annual change in SHS	Mean (SD)	2.7 (8.7) − 8–52	1.7 (2.5) 0–12	1.6 (1.5) 0–4	1.3 (1.5) 0–4
		range				
	No. (percent) of RRP		3 (7.7)	1 (3.4)	0 (0)	0 (0)
Incomplete sustained remission	Annual change in SHS	Mean (SD)	2.7 (3.9) − 2–14	2.3 (8.2) − 8–52	3.5 (8.6) − 8–52	3.2 (9.0) − 2–52
		range				
	No. (percent) of RRP		9 (20.9)	4 (9.5)	9 (19.1)	3 (8.3)
None remission	Annual change in SHS	Mean (SD)	5.5 (10.3) − 6–58	5.1 (9.1) − 6–58	4.8 (9.1) − 6–58	4.2 (8.3) − 8–58
		range				
	No. (percent) of RRP		20 (32.3)	29 (32.2)	25 (29.8)	31 (27.7)

Radiographic progression was assessed by annual change in Sharp/van der Heijde score (SHS) during follow-up period. RRP (rapid radiographic progression) was defined as 5 or more unit change in SHS per year. Complete sustained remission was defined as maintain rate of 100%. Nearly sustained remission was defined as maintain rate of 50% and more. Incomplete sustained remission was defined as maintain rate of less than 50%. None remission was defined as maintain rate of 0%

In conclusion, this study clearly demonstrated that sustained clinical remission contributes to reduced radiological progression in RA.

### Bone and cartilage regeneration in rheumatology clinical practice

The results described above clearly show that not only achieving remission but also maintaining remission is crucial for preventing joint destruction. However, is it possible to achieve bone regeneration using any type of treatment? And if so, how?

Historically, it was the universal consensus in clinical rheumatology that joint destruction could not be reversed by any kind of treatment. In other words, once any part of the joint was destroyed, nothing could be done for the joint other than trying to prevent further destruction. This was the main reason why practitioners were eager to start aggressive treatment before any joint destruction was observed. This concept still holds mostly true in the current medical situation. However, even before the advent of highly effective treatments such as bDMARDs and targeted synthetic DMARDs (tsDMARDs), bone regeneration or healing was observed in a small proportion of RA patients. In a pioneering case report, Rau and Herborn described the healing of erosive changes in six RA patients who were treated with methotrexate and/or gold sodium thiomalate (GST) [26]. Although Rau and colleagues repeatedly reported such cases, it was still considered that such healing was very rare [26–28].

However, the dramatic results of treatment with bDMARDs have gradually changed the entire basis of the treatment strategy for RA and practitioners’ frame of mind in terms of bone destruction and regeneration. The 2005 article by Ikari and Momohara clearly showed that methotrexate can induce bone regeneration [29], and many practitioners have seen such cases repeatedly in their routine clinical practice. Indeed, some of the first randomized controlled studies using bDMARDs reported negative average values for joint destruction after a certain period of treatment [30–32], which means that the majority of patients who used bDMARDs experienced bone regeneration in one way or another.

However, critics or doubters claimed that such regeneration was seen only in small joints such as the proximal interphalangeal, metacarpophalangeal, or metatarsophalangeal joints in the fingers or toes. Indeed, most case reports have shown bone regeneration only of small joints. However, Momohara’s case report clearly showed that even a large joint (hip joint) can achieve cartilage regeneration, or at least the reappearance of the joint space, with an effective treatment such as bDMARDs [33]. After this pioneering case report, clinical studies including a case series have described bone regeneration of large joints [34]. As a result, a Japanese research group has established a radiological change score called the ARASHI change score, which incorporates the improvements in bone quality, joint space narrowing, joint conformity, and the disappearance of bone erosion and joint surface destruction [35]. Unfortunately, it is still the case that joint regeneration in the large joints is relatively rare. Seki and Matsushita have shown that, once joint damage has been detected, damage in the ankle joint tends to be less progressive but joint destruction in the hip and knee joints is likely to progress even following a good response to anti-tumor necrosis factor alpha treatment [36–38]. Therefore, it remains largely unresolved whether a large joint can regenerate, and if so, how it regenerates. Other unresolved issues are the nature of the predictors of large-joint regeneration, and how we can intervene to stimulate regeneration or, at least, to prevent the progression of large-joint destruction.

### Is it possible to induce joint regeneration by surgical management?

Most therapeutic strategies for prevention or regeneration of joint destruction are based on the use of effective medications. However, oral or intravenous medication has an effect on the whole body, i.e., it diffuses throughout the whole body and may therefore be less effective in a particular joint. One of the potent options for treating a particular joint is intra-articular injection. Indeed, several studies indicate that intra-articular injection of steroid is highly effective and comparable to bDMARDs for the alleviation of disease activity [39]. For example, a preliminary report showed that in osteoarthritis, intrajoint injection of bDMARDs can achieve a better response than injection of hyaluronan [40]. Moreover, a recent study showed that intra-articular glucocorticoids in combination with methotrexate can induce bone regeneration in some cases of RA, although this response is relatively rare [41]. Future investigations should determine which patients should receive intra-articular steroid injection and its optimal timing.

Another possible approach to this issue is surgical intervention. Joint regeneration, especially cartilage regeneration, has been widely investigated over the last three decades. We recently published a report of scaffoldless hyaline cartilaginous tissue derived from induced pluripotent stem cells [42]. However, despite committed, long-term efforts worldwide, clinically useful hyaline cartilage regeneration has not yet been achieved. To overcome this highly problematic threshold to achieving joint regeneration, the most plausible treatment strategy is to induce or assist the patient’s own ability to regenerate bone and cartilage. In the case of inflammatory arthritis such as RA, the reduction of synovitis or the surgical removal of inflammatory synovia is one plausible option. We have experienced one such case in the past.

**Fig. 6:**
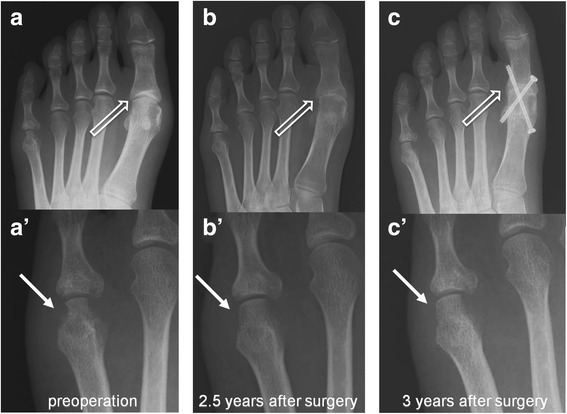
**a**–**c**, **a**′–**c**′ Bone regeneration after synovectomy of the 5th metatarsophalangeal joint of the left foot. The left panel shows the preoperative radiograph of the left foot. Bone erosion was seen in the 5th metatarsal head. The middle panel shows the reappearance of the 5th metatarsal head 2.5 years after synovectomy of the joint. Joint space narrowing appeared at the 1st metatarsophalangeal joint. The right panel shows consolidation of the 5th metatarsal head. The 1st metatarsophalangeal joint was fixed

The patient, a 21-year-old woman, presented to our hospital with pain and a soft tissue mass on her left fifth metatarsophalangeal joint. Radiographic and magnetic resonance imaging showed a tumor-like mass with destruction of the joint (Fig. 6a′, white arrow), which was reported as being suggestive of a benign tumor. At surgery, synovia-like tissues were observed that had migrated into the bare area of this joint. This lesion was successfully removed, and histological analysis confirmed synovitis, suspicious of RA. The patient gradually developed polyarticular synovitis, and methotrexate was started 3 years later. Surprisingly, bone repair of this joint was achieved 1 year after surgery and was maintained without recurrence of synovitis for more than 5 years (Fig. 6b′, c′, white arrows). In contrast, her left first metatarsophalangeal joint gradually developed destructive changes (Fig. 6a, b, white thick arrow), which led to surgical arthrodesis 3 years later (Fig. 6c, white thick arrow).

Some authors recommend surgical synovectomy for patients with RA who do not experience substantial pain relief in response to medications. By removing all synovial tissues, synovectomy can diminish local pain and swelling, but bone repair of damaged joints has not been expected. This case provides evidence that synovectomy can induce bone repair of a damaged joint in a patient with early RA. To the best of our knowledge, this is the very first report that synovectomy indeed stimulated joint regeneration. Pinder previously reported that synovectomy with drilling of areas of articular cartilage loss showed cartilage regeneration and relief of symptoms [43]. However, since then, no other report has shown similar results by any surgical procedures. The reason of his success may be that he probably conducted this procedure in patients with very low disease activity. But the regeneration potential of the joint should be paid with full attention even in RA patients in the current medication.

Also, the molecular mechanisms of how the regeneration occurs have been investigated and proposed, which has attracted huge attention from basic researchers. Several review articles recently summarized the proposed mechanism of bone remodeling in RA that proinflammatory cytokines such as TNF alpha stimulates the production of DKK-1 family and soluble frizzled related protein, suggesting that inhibition of such cytokines downregulates those proteins and revives bone formation processes [44, 45]. Wehmeyer et al. recently lay stress on the importance of stromal cells which release Wnt antagonists such as sclerostin and DKK-1 under inflammatory conditions [44, 46]. Taken together, blocking proinflammatory cytokines or removal of synovial tissues producing such cytokines can regain the balance of bone resorption and formation and can stimulate bone regeneration. Adding to the suppression of proinflammatory cytokines or cells, suppression of Wnt antagonists or stromal cells may be a potent therapeutic option in the future.

### Future perspectives

In inflammatory arthritis, synovitis causes bone and cartilage destruction as described above. One of the most crucial requirements for regeneration of the destroyed joint is alleviation of synovitis. This can be achieved by use of adequate medication as soon as possible after the diagnosis of the disease. When the joint has the ability to regenerate the destroyed bone and/or articular cartilage, self-regeneration should occur after alleviation of the synovitis. However, regenerative medicine will have a crucial role in treatment when this ability is lost, or when the destruction is too severe to be overcome. Although it is still uncertain what kind of treatment options will be available in routine clinical practice, regenerative medicine should be able to rescue the damaged joint using potent cell therapies.

## Conclusions

To prevent joint destruction in inflammatory arthritis such as RA, the universal consensus is to treat, to alleviate synovitis and to achieve clinical remission. Our study shows that maintaining remission is also crucial to prevent the progression of joint destruction. Although regeneration of the damaged joint has been considered to occur very rarely, accumulating evidence shows that it can actually occur in routine clinical practice after strong inhibition of synovitis with highly potent medications. Two potent options other than oral or intravenous medication for inducing joint regeneration in a particular joint would be intra-articular steroid injection and synovectomy. In the future, regenerative medicine could play a crucial role in inducing regeneration of damaged joints after synovitis is effectively inhibited when self-regeneration cannot overcome severe destruction.

## Abbreviations

ACR: American College of Rheumatology
bDMARD: Biological DMARD
CDAI: Clinical disease activity index
csDMARD: Conventional synthetic DMARD
DAS28: Disease activity score involving a 28 joints
DAS44: Disease activity score based on 44 joints
DMARD: Disease-modifying anti-rheumatic drug
EGA: Global assessments of disease activity by evaluators
ESR: Erythrocyte sedimentation rate
EULAR: The European League Against Rheumatism
HAQ-DI: The Health Assessment Questionnaire disability index
KURAMA: The Kyoto University Rheumatoid Arthritis Management Alliance
PGA: Global assessments of disease activity by patients
RA: Rheumatoid arthritis
RF: Rheumatoid factor
RRP: Rapid radiographic progression
SDAI: Simplified disease activity index
SHS: Sharp/van der Heijde score
SJC28: Swollen joint count based on assessment of 28 joints
T2T: Treat to target
TJC28: Tender joint count based on assessment of 28 joints
tsDMARD: Targeted synthetic DMARD
VAS: Visual analogue scale
